# Highly Adsorptive Au-TiO_2_ Nanocomposites for the SERS Face Mask Allow the Machine-Learning-Based Quantitative Assay of SARS-CoV-2 in Artificial Breath Aerosols

**DOI:** 10.1021/acsami.2c16446

**Published:** 2022-11-30

**Authors:** Charles S. H. Hwang, Sangyeon Lee, Sejin Lee, Hanjin Kim, Taejoon Kang, Doheon Lee, Ki-Hun Jeong

**Affiliations:** † Department of Bio and Brain Engineering, Korea Advanced Institute of Science and Technology (KAIST), 291 Daehak-ro, Yuseong-gu, Daejeon 34141, Korea; ‡ Bionanotechnology Research Center, Korea Research Institute of Bioscience and Biotechnology (KRIBB), 125 Gwahak-ro, Yuseong-gu, Daejeon 34141, Korea; § School of Pharmacy, Sungkyunkwan University, Suwon 16419, Korea; ∥ KAIST Institute for Health Science and Technology (KIHST), Korea Advanced Institute of Science and Technology (KAIST), 291 Daehak-ro, Yuseong-gu, Daejeon 34141, Korea

**Keywords:** SARS-CoV-2, surface-enhanced Raman spectroscopy, breath biopsy, machine-learning, plasmonics, nanocomposite

## Abstract

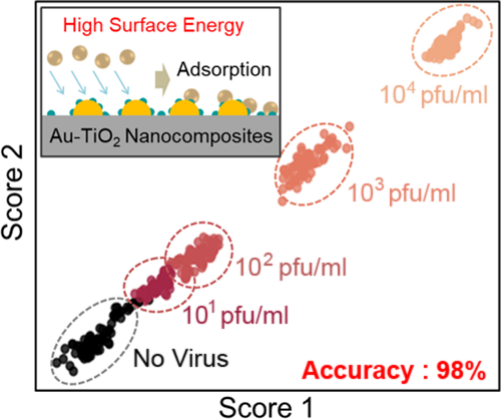

Human respiratory aerosols contain diverse potential biomarkers for early disease diagnosis. Here, we report the direct and label-free detection of SARS-CoV-2 in respiratory aerosols using a highly adsorptive Au-TiO_2_ nanocomposite SERS face mask and an ablation-assisted autoencoder. The Au-TiO_2_ SERS face mask continuously preconcentrates and efficiently captures the oronasal aerosols, which substantially enhances the SERS signal intensities by 47% compared to simple Au nanoislands. The ultrasensitive Au-TiO_2_ nanocomposites also demonstrate the successful detection of SARS-CoV-2 spike proteins in artificial respiratory aerosols at a 100 pM concentration level. The deep learning-based autoencoder, followed by the partial ablation of nondiscriminant SERS features of spike proteins, allows a quantitative assay of the 10^1^–10^4^ pfu/mL SARS-CoV-2 lysates (comparable to 19–29 PCR cyclic threshold from COVID-19 patients) in aerosols with an accuracy of over 98%. The Au-TiO_2_ SERS face mask provides a platform for breath biopsy for the detection of various biomarkers in respiratory aerosols.

## Introduction

Respiratory airborne particulates from human breath contain vital information on the current health status of an individual.^1,2^ Different forms of such particulates, i.e., aerosols and volatile organic compounds (VOCs), contain potential biomarkers for a wide spectrum of diseases including viruses, asthma, cancers, and neurodegenerative disorders.^3−8^ In particular, respiratory aerosols from human breath have attracted significant interest with the recent global outbreak of the 2019 coronavirus disease (COVID-19) pandemic caused by severe acute respiratory syndrome coronavirus-2 (SARS-CoV-2).^9−11^ With the first report in the late December of 2019 in Wuhan, China, there are over 500 million confirmed cases globally as of April 2022.^12^ Both the World Health Organization and the United States Centers for Disease Control and Prevention have confirmed that COVID-19 is principally transmitted through respiratory aerosols.^12,13^ Combined with the high proportion of asymptomatic patients reaching up to 40% of the total confirmed patients, the reproduction number of COVID-19 has been reported to be 4–20 times greater than that of seasonal flu.^14^ However, the current gold standard for the diagnosis of such large-scale COVID-19 patients is still limited to nucleic acid detection by amplification,^15,16^ where the examination process takes up to several hours under the supervision of highly qualified medical personnel. COVID-19 face masks have recently been demonstrated that diagnose the collected viruses on a cellulose matrix;^17,18^ yet, these also require time-consuming post-extraction processes for a polymerase chain reaction. In contrast, a comprehensive and direct examination of breath aerosols has a high potential for the rapid diagnosis of COVID-19 but has been relatively unexplored for such diagnostic applications.

Recently, surface-enhanced Raman spectroscopy (SERS) has demonstrated the detection and analysis of aerosols for diverse air monitoring applications by utilizing the electromagnetic “hotspots” of plasmonic nanostructures.^4,19−22^ Unlike conventional aerosol detection methods such as physical impactor and evaporative light scattering detection, SERS serves as a cost-effective alternative for rapid, sensitive, and quantitative analysis of chemical fingerprints within the aerosols. However, plasmonic SERS substrates often show some technical limitations in detecting respiratory breath aerosols due to a low Weber number of an aerosol, i.e., the ratio of the aerosol’s momentum to the surface tension.^23^ In other words, the fast emission velocity of such aerosols (2–10 m/s)^24^ substantially deters effective adsorption onto a substrate. While conventional SERS substrates with assorted nanostructures^25−29^ and nanoparticles^30,31^ display highly packed geometries for enhanced electromagnetic hotspots, they often result in low surface energy that is inefficient for aerosol adsorption. As a result, hotspot-rich SERS substrates with high surface energies are still in need of efficient capture and facile preconcentration of respiratory aerosols.

## Results and Discussion

**Figure 1:**
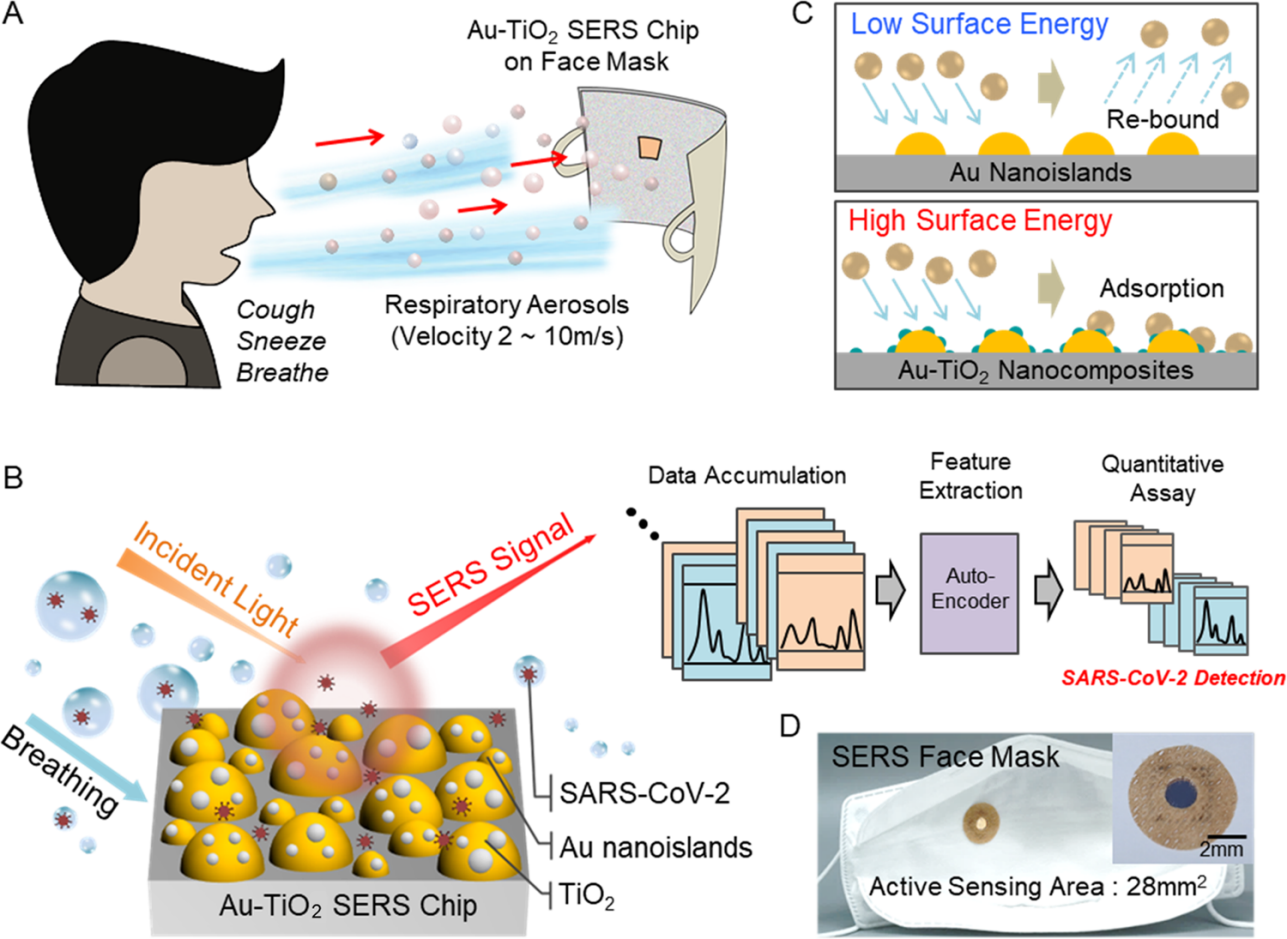
Schematic illustration of SARS-CoV-2 detection from respiratory breath aerosols using the Au-TiO_2_ SERS chip on a face mask. (a) Oronasal aerosols emitted upon respiratory actions are easily and directly collected onto the SERS chip. (b) Machine-learned SERS detection of SARS-CoV-2 aerosols. The measured SERS signals are quantitatively classified using an ablation-assisted autoencoder. (c) Aerodynamic behavior of aerosols impacting on a solid substrate with different surface energies. Au-TiO_2_ nanoislands with high surface energies efficiently adsorb the low-volume and high-velocity respiratory aerosols. (d) Optical image of the SERS face mask as an application example. The Au-TiO_2_ SERS chip was attached to the inner cloth of a KF94 mask with a medical plaster.

Here, we report a SERS face mask for the label-free detection of the aerosolized SARS-COV-2 virus using gold-titanium dioxide (Au-TiO_2_) nanocomposites. The face mask features a highly adsorptive Au-TiO_2_ nanocomposite SERS chip on the inner cloth of a filtering facepiece respirator (Figure 1a). Human breath aerosols are oronasally released during respiratory action such as breathing, coughing, sneezing, or speaking and are continuously preconcentrated onto the SERS chip inside the respirator. Au-TiO_2_ nanoislands on a quartz substrate exhibit high aerosol adsorption and dense electromagnetic hotspots, allowing the rapid, facile, and quantitative SERS detection of the SARS-CoV-2 virus (Figure 1b). An autoencoder neural network is further employed for the accurate classification of the SARS-CoV-2 virus at various concentrations. The aerodynamic behavior of aerosols impacting a surface mainly depends on the surface energy (Figure 1c). For instance, respiratory aerosols with an average velocity of 2–10 m/s^24^ simply rebound upon impact with conventional Au nanoislands due to low surface energy.^23^ In contrast, an ultrathin film of TiO_2_ nanoclustered in the Volmer–Weber mode on the Au nanoislands significantly increases the surface energy (contact angle, θ = 20–30°), allowing the efficient adsorption of the respiratory aerosols. The Au-TiO_2_ SERS chip with an active sensing area of 28 mm^2^ was diced and attached to a commercial KF94 respirator with a round medical plaster bandage (Figure 1d).

**Figure 2:**
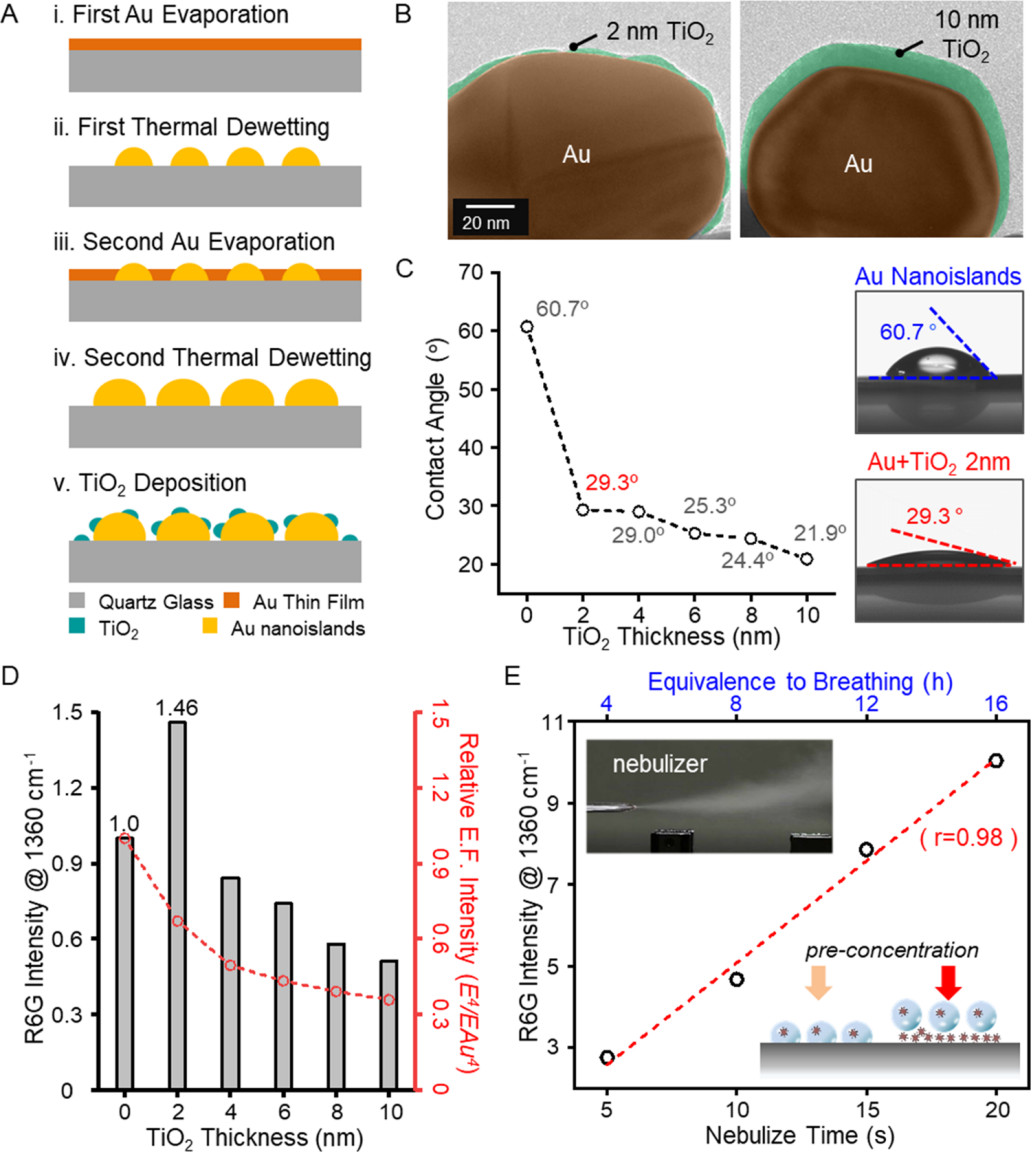
Highly adsorptive Au-TiO_2_ nanocomposites substrate for aerosol preconcentration and enhanced SERS signals. (a) Nanofabrication process employs repeated solid-state dewetting of Au thin films for high-density EM hotspot generation. A thin layer of TiO_2_ is then thermally evaporated to obtain Au-TiO_2_ nanocomposites. (b) FE-TEM images of the fabricated Au-TiO_2_ nanoislands for 2 nm (left) and 10 nm (right) thin films of TiO_2_. (c) Contact angles for various thicknesses of TiO_2_ on Au nanoislands. The 2 nm thick TiO_2_ thin film substantially increases the surface energy. (d) Comparison of measured SERS intensity of R6G aerosols and calculated E-field intensities for different thicknesses of TiO_2_. The Au-TiO_2_ substrate with 2 nm TiO_2_ exhibits 47% increased SERS intensity of R6G aerosols at 1360 cm^–1^ due to highly adsorptive surfaces efficiently adsorbing nebulized R6G aerosols. (e) Preconcentration of 100 nM R6G aerosols using a nebulizer. The SERS intensity of R6G at 1360 cm^–1^ linearly increases with respect to the preconcentration times.

The enhanced surface energy of the Au-TiO_2_ nanocomposites is crucial for the highly sensitive SERS detection of respiratory aerosols. The nanofabrication of Au-TiO_2_ nanocomposites exploits a facile two-step process including repeated thermal dewetting of Au thin films and thermal evaporation of TiO_2_ (Figure 2a). The hotspot-rich Au nanoislands were fabricated at repeated dewetting of 6 + 6 nm thick Au thin films, showing a 21% increase in average size, 72% decrease in the interparticle distance, and 5-fold E-field enhancement (Figure. S1), compared to single dewetting of a 12 nm thick Au thin film. See the Methods section for the repeated thermal dewetting of Au nanoislands, geometric characterization of Au nanoislands, and FDTD calculation of E-field enhancements. A 2 nm thick TiO_2_ film was evaporated in the Volmer–Weber mode onto the as-fabricated Au nanoislands to form Au-TiO_2_ nanocomposites. The geometry and the composition of Au-TiO_2_ nanocomposites were characterized using a field-emission transmission electron microscope (300 keV FE-TEM, Tecnai G^2^ F30 S-TWIN, FEI) equipped with energy-dispersive X-ray (EDX) (Figures 2b and S2). The ultrathin TiO_2_ film forms nanoclusters on Au nanoislands, while TiO_2_ of larger thickness leads to uniform thin-film formation.^32,33^ The contact angle of a water droplet was then measured to compare the surface energy of Au-TiO_2_ substrates with different TiO_2_ thicknesses ranging from 0, 2, 4, 6, 8, and 10 nm (Figure 2c). While simple Au nanoislands without TiO_2_ show a contact angle of 60°, even a slight addition of 2 nm thick TiO_2_ significantly reduces the contact angle to 29°. The measured SERS signals of nebulized 10^–7^ M rhodamine 6 g (R6G) also show that the Au-TiO_2_ substrate efficiently captures aerosols from the increased surface energy. The E-field intensities of the Au-TiO_2_ nanocomposites were calculated using the FDTD method and compared with the observed SERS peak intensity of nebulized R6G aerosols at 1360 cm^–1^ (Figures 2d and S3). See the Methods section for the FDTD calculation of E-field enhancements. The relative E-field intensity ratio (E^4^/E_Au_^4^) rapidly decays to 0.67, 0.49, 0.43, 0.39, and 0.36 for increasing TiO_2_ thickness of 2, 4, 6, 8, and 10 nm because the TiO_2_ thin films behave as a dielectric gap spacer.^34^ However, the SERS intensity ratios at 1360 cm^–1^ are observed as 1.47, 0.81, 0.74, 0.58, and 0.51 with respect to that of simple Au. This phenomenon is explained by the highly adsorptive Au-TiO_2_ substrate efficiently capturing more aerosols that compensates for the SERS intensity decay. In particular, the 2 nm TiO_2_ thin film evaporated as nanoclusters on Au allows direct contact of the target analytes in the aerosol to the plasmonic hotspots, exhibiting substantially enhanced SERS intensity. The preconcentration of aerosols on the face mask was further investigated by measuring the SERS signals of 10^–7^ M R6G for increasing nebulizing times (Figure 2e). The low-volume aerosols dry quickly, allowing for the continuous accumulation of target analytes within the aerosols. The SERS intensity at 1360 cm^–1^ linearly increases as the preconcentration time of the aerosols changes from 5, 10, 15, and 20 s. Note that the collection of aerosols nebulized during five seconds is equivalent to a four-hour preconcentration of aerosols from respiration.^35^

**Figure 3:**
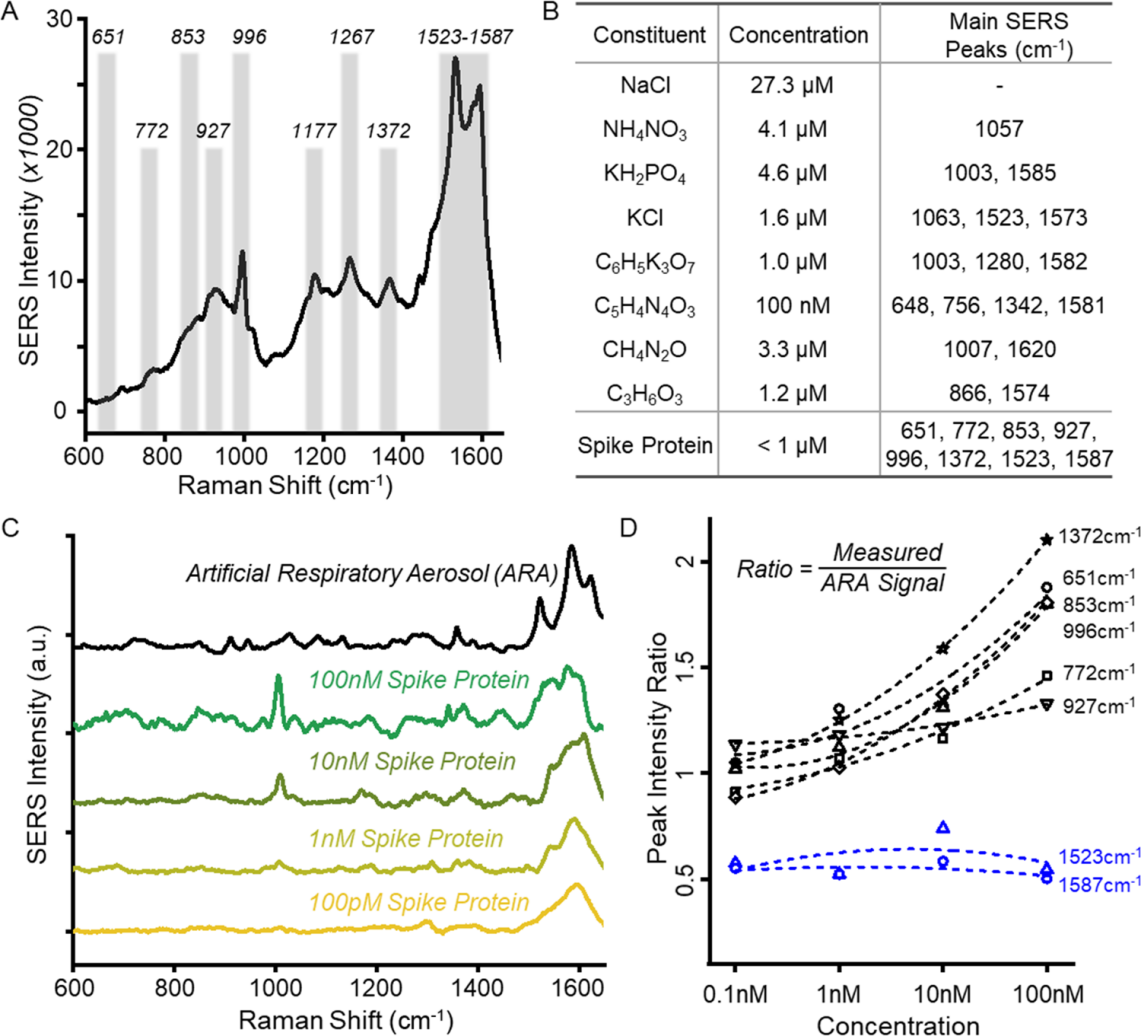
SERS measurements of SARS-CoV-2 spike proteins in artificial respiratory aerosols. (a) SERS signals of SARS-CoV-2 spike protein aerosols adsorbed on Au-TiO_2_ nanocomposite substrates. The characteristic Raman peaks of the spike proteins are clearly observable at 651, 772, 853, 927, 996, 1177, 1267, 1372, 1523, and 1587 cm^–1^. (b) Composition of artificial respiratory aerosols (ARA) and measured SERS peaks. Raman bands of the spike proteins at 1523 and 1587 cm^–1^ overlap with those of artificial respiratory aerosols. (c) SERS signals at various concentrations of the spike proteins in ARA. (d) SERS signal intensity ratios at various characteristic Raman bands of the spike proteins were calculated using ARA as the reference. Some Raman bands, i.e., 1523 and 1587 cm^–1^ Raman bands, are shadowed by the reference signals, while most others provide quantitative fingerprints of SARS-CoV-2 spike proteins in ARA.

Characteristic SERS peaks of SARS-CoV-2 spike proteins in artificial respiratory aerosols were characterized using the Au-TiO_2_ substrates. The SARS-CoV-2 is surrounded by protruding spike proteins, which serve as a primary biomarker for immunoassays in the receptor recognition and membrane fusion process.^36^ Several characteristic SERS peaks in 1 μM SARS-CoV-2 spike proteins are observed including CH_2_ rocking of phenylalanine at 651, 772, and 996 cm^–1^, CH_2_ rocking of tryptophan at 852 cm^–1^, NH_3_ rocking of histidine at 1177 cm^–1^, CH_2_ wagging of L-arginine at 1267 cm^–1^, C-N stretching and amide III band at 1372 cm^–1^, and CH_2_ deformation and NH bending of tryptophan and phenylalanine at 1523 and 1587 cm^–1^ (Figure 3a).^37^ Note that the SERS signals of SARS-CoV-2 spike proteins show high selectivity to other viral proteins.^38,39^ Also see Figure S4 for SERS signals of the spike proteins with different concentrations ranging from 1 μM to 100 pM. Next, the SARS-CoV-2 spike proteins with concentrations ranging from 100 nM to 100 pM were mixed in an artificial respiratory solution and nebulized onto the SERS substrate for the emulation of the human oronasal emission. The chemical constituents,^40^ concentrations, and observed SERS peaks of the artificial respiratory aerosols (ARA) are respectively summarized in Figures 3b and S5. The ARA samples were prepared by adding spike proteins of different concentrations to the stock artificial respiratory solution. See Methods for the emulation of oronasal aerosols and artificial respiratory solutions. Strong SERS bands are observed in ARA at the 1400–1600 cm^–1^ range, resulting from various chemicals such as potassium citrate, lactic acid urea, etc. As a result, the SERS signals of the spike protein in ARA (Figure 3c) are partially different from that of Figure 3a, particularly at the 1400–1600 cm^–1^ range, due to signal interference and different adsorption kinetics in complex mixtures caused by ARA.^41^ The characteristic SERS peaks of the SARS-CoV-2 spike proteins in Figure 3a were then quantitatively compared by calculating the SERS peak intensity ratios depending on the spike protein concentration, with respect to ARA as the reference (Figure 3d). While the signal intensity ratio of nondiscriminant peaks (1523 and 1587 cm^–1^) remains relatively constant, all other characteristic peaks are highly discriminant and increase for a higher concentration of the SARS-CoV-2 spike protein in ARA.

**Figure 4:**
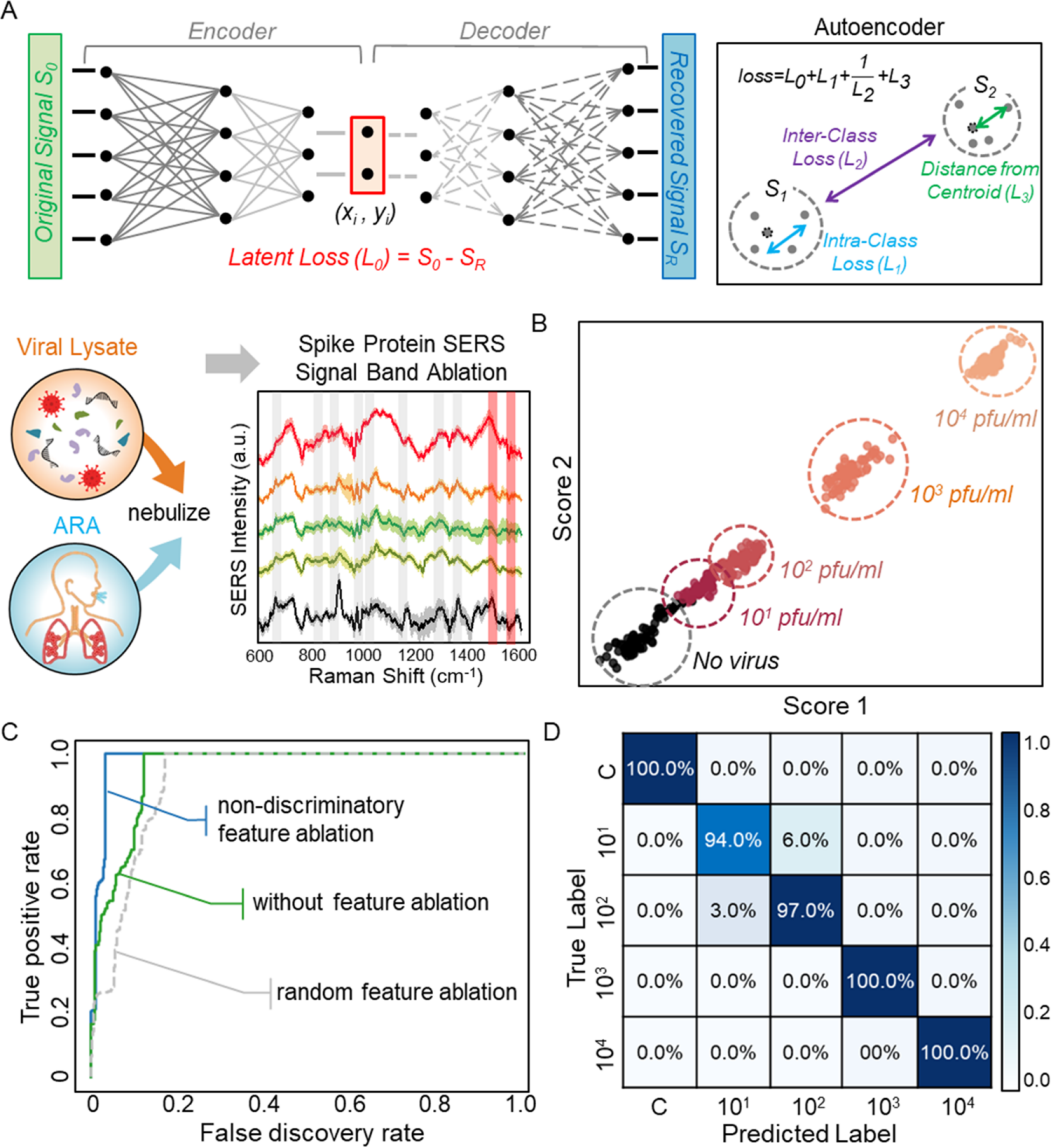
Deep learning-based quantitative SERS assay for label-free SARS-CoV-2 lysates in ARA. (a) Ablation-assisted autoencoding. The nasopharyngeal SARS-CoV-2 lysates were nebulized with ARA for initial SERS measurements, followed by the ablation of the low-importance SERS features of spike proteins in ARA. Then, an autoencoder was used to quantitatively classify the SERS signals of the viral lysates (10^1^–10^4^ pfu/mL) in ARA. The 2D latent space scores were optimized with hyperparameters including latent loss (*L*_*0*_), intraclass (*L*_*1*_), interclass (*L*_*2*_), and distances from centroids (*L*_*3*_). (b) Two-dimensional latent scores of SERS signals of SARS-CoV-2 lysates with different concentrations. The classified clusters show a strong diagonal alignment with SARS-CoV-2 concentrations. (c) Area under the receiver operating characteristic curve (ROC) for the ablation-assisted autoencoder algorithm. (d) Confusion matrix of the autoencoder algorithm exhibiting the average prediction accuracy of over 98% from the label-free SERS detection of SARS-CoV-2 lysates.

An ablation-assisted autoencoder with a logistic regression model was further employed for a quantitative assay of SARS-Cov-2 lysate in aerosols. The autoencoder algorithm allows characteristic feature extraction by reducing the dimensions of input signals into low-dimensional features in the latent space.^42,43^ The autoencoder-based dimensionality reduction model was trained in a supervised manner to minimize the total loss function including latent loss, interclass, intraclass, and distances from centroids^44^ (Figure 4a). See the Methods section for training the autoencoder model. The initial SERS signals of SARS-CoV-2 lysate aerosols in artificial respiratory solutions were acquired for different concentrations ranging from 0, 10^1^, 10^2^, 10^3^, and 10^4^ pfu/mL at five different positions on the Au-TiO_2_ substrates (Figures 4a and S6). The SERS signals mapped onto the two-dimensional (2D) latent space via the autoencoder reveal that the individual classes are well-clustered and diagonally aligned depending on the lysate concentration (Figure 4b). This allows the logistic regression model to be used as a prediction model for the highly accurate quantitative assay of target molecules in the complex solution, i.e., artificial respiratory aerosols, that supersede the conventional chemometrics such as principal component analysis. The ablation of the nondiscriminant Raman features of spike proteins in ARA further substantially increases the classification accuracy of the prediction model (Figure 4c). The two nondiscriminant SERS features of the spike proteins in ARA, i.e., 1532 and 1587 cm^–1^ bands, were ablated based on Figure 3. Data sets with the random ablation of the same length of vectors were also generated for comparison. Each of the data sets with nondiscriminatory ablation, random ablation, and without ablation was then trained using the autoencoder-based dimensionality reduction model, followed by logistic regression models. The receiver operating characteristic (ROC) curve explicitly demonstrates that the ablation of the 1532 and 1587 cm^–1^ SERS peaks provides 7.6% higher classification accuracy compared to nonablated SERS signals. The confusion matrix of the ablation-assisted autoencoder model indicates that the SARS-CoV-2 lysates adsorbed on the Au-TiO_2_ nanocomposite SERS substrate exhibit over 98% classification accuracy for concentrations ranging from 10^1^–10^4^ pfu/mL (Figure 4d). Note that typical COVID-19 patients demonstrate an average cyclic threshold value of 27 during PCR testing,^45^ which corresponds to 10^2^–10^3^ pfu/mL concentration of the SARS-CoV-2 virus.^46^ As a result, the highly accurate assay of the SARS-CoV-2 lysates down to 10^1^ pfu/mL infers the successful diagnosis of COVID-19 from respiratory aerosols.

## Conclusions

To conclude, this work has successfully demonstrated a highly adsorptive Au-TiO_2_ nanocomposite-based SERS face mask for the SERS detection of SARS-CoV-2 in artificial breath aerosols. Nanoclustered TiO_2_ on Au nanoislands efficiently adsorbs respiratory aerosols and shows 47% enhanced SERS signals compared to conventional Au nanoislands. The SERS chip inside a face mask further exhibits preconcentration of low-volume respiratory aerosols. The autoencoder prediction model with a modified loss function demonstrates the quantitative assay of SARS-CoV-2 lysates with 98% accuracy by ablating nondiscriminant SERS peaks of spike proteins. This Au-TiO_2_ SERS face mask provides a rapid, robust, and facile screening method for the pre-emptive diagnosis of COVID-19 and a biosensing platform for breath biopsy, detecting various disease biomarkers in respiratory aerosols.

## Methods

### Repeated Thermal Dewetting of Au Nanoislands

First, a 6 nm thick Au thin film was thermally evaporated onto a 4-inch quartz wafer at a constant rate of 0.5 Å/s. The Au thin film was then thermally dewetted inside a box furnace (Lindberg/Blue M, Moldatherm Box Furnace) for one hour at 700 °C to form large-area Au nanoislands with high adhesion to glass. The target temperature was steadily increased from the room temperature with ramp-up and ramp-down rates of 20 and 5 °C/min, respectively. The above procedure was repeated once more to form closely packed and enlarged Au nanoislands for highly uniform and substantially increased SERS signals.^47^

### Geometric Characterization of Au Nanoislands

The SEM images were directly converted into binary images. The radius and interparticle distances of the Au nanoislands after single and repeated dewetting were calculated with ImageJ software, assuming that Au nanoislands are periodic arrays with uniform sizes.

### FDTD Calculation of E-Field Intensities

The three-dimensional finite-difference time-domain (FDTD) simulation was performed to calculate the E-field enhancement of the nanoislands by directly importing the two-dimensional SEM images. The Au nanoislands were assumed to have a thickness of 58 nm on the dielectric surface (*n* = 1.4) with a uniform thin-film TiO_2_ layer on top of the nanoislands with respect to the target thickness. The TiO_2_ layer was disabled for the E-field comparison between single and repeated thermal dewetting. The E-field intensity was monitored at a 633 nm wavelength region positioned at 20 nm above the substrate. The average E-field intensity for the region of interest was utilized for the calculation of the relative E-field intensity (E_nanocomposite_^4^/E_Au_^4^). (Release: 2019a r6, Version 8.21.1933)

### Emulation of Oronasal Aerosols and Artificial Respiratory Solutions

The oronasal emission of human respiratory aerosols was emulated using a commercial nebulizer (Teledyne CETAC Technologies) (Figure 2d). The carrier gas at a 200 sccm flow rate was controlled using a mass flow controller (MKS Instruments), while the Au-TiO_2_ nanocomposite SERS substrate was positioned 25 cm away from the nebulizer’s end in a fume hood. The average size of the nebulized aerosol is <10 μm. The nebulizing time was 10 seconds. The artificial respiratory solution was prepared to emulate respiratory aerosols from human breath. SARS-CoV-2 spike proteins with respective concentrations were diluted in a stock solution with final concentrations of 27.3 μM NaCl, 4.1 μM NH_4_NO_3_, 4.6 μM KH_2_PO_4_, 1.6 μM KCl, 1.0 μM C_6_H_5_K_3_O_7_, 0.1 μM C_5_H_4_N_4_O_3_, 3.3 μM CH_4_N_2_O, and 1.2 μM C_3_H_6_O_3_ in deionized water.

### SARS-CoV-2 Lysate Preparation

The SARS-CoV-2 (BetaCoV/Korea/KCDC03/2020) was provided by the National Culture Collection for pathogens, which is operated by the Korea National Institute of Health. The virus was carefully cultured in a biosafety level 3 laboratory at the Korea Research Institute of Bioscience and Biotechnology (KRIBB) and heated with the lysis buffer.^48^ The stock SARS-CoV-2 lysates were then diluted to concentrations ranging from 10^1^–10^4^ pfu/mL and mixed in artificial respiratory solution prior to nebulizing. The control sample in Figure 4 refers to an artificial respiratory solution mixed with the culture media and lysis buffer without any viral lysates.

### SERS Signal Measurement of Nebulized R6G Aerosols

The 10^–7^ M R6G was nebulized for 10 s, and the SERS signals were measured using a benchtop spectrometer equipped with a CCD camera and 50× objective lens (MicroSpec 2300i, Princeton Instruments) under excitation of 5 mW 633 nm HeNe laser. The acquisition time was 1 s.

### Training the Autoencoder Model

First, five SERS spectra measured at different positions on the SERS substrate were obtained for different concentrations of aerosolized SARS-CoV-2 lysates in an artificial respiratory solution (control, 10^1^, 10^2^, 10^3^, 10^4^ pfu/mL). The measured SERS spectra with a vector length of 1096, containing the signal intensities for the Raman shift range of 600–1580 cm^–1^, were used for the quantitative analysis of SARS-CoV-2 lysates. The number of measured SERS data was augmented to improve the performance and the robustness of the prediction model and to prevent overfitting by adding random noise and applying the synthetic minority oversampling technique (SMOTE) algorithm provided by the Python package.^49^ Twenty synthetic data with random noise were generated from five measured SERS data sets, followed by generating 80 additional synthetic data from the SMOTE algorithm with a shrinkage value of 1.8 for each concentration class of SARS-CoV-2 lysates. The amplitude of noise for a wavenumber was randomly determined between ±1/5 of the original SERS value. One hundred vectors were acquired for each SARS-CoV-2 lysate concentration. The total 500 vectors, resulting from five different concentrations, were finally utilized for the autoencoder training. The model consists of a symmetric encoder and decoder with 1069 input nodes and two fully-connected layers, each consisting of 256 and 36 nodes and two latent nodes. The batch size and epoch were manually set as 32 and 40, respectively, with a 1e^–4^ learning rate and 1e^–4^ weight decay. Other hyperparameters for the linearizing autoencoder, such as weights of loss, are heuristically determined through the experiment. An entirely different data set of 500 test data was generated for validation and evaluation after the training—475 synthetic vectors were generated using the aforementioned protocol of generating synthetic training data and combined with 25 measured SERS data. Fifty data points on both sides of the nondiscriminant SERS bands of the SARS-CoV-2 spike were set to zero during the training of the ablation-assisted autoencoder. The same number of wavenumbers at random points was set to zero as a control set. The concentrations of the SARS-CoV-2 lysate data were classified by the logistic regression of the two-dimensional latent features from the autoencoder. The ROC curve is calculated during the classification and macro-averaged for every label.

## Supporting Information Available

SERS EF comparison between single and repeatedly dewetted Au nanoislands (Figure S1); EDX elemental analysis of Au-TiO_2_ nanocomposites (Figure S2); SERS intensity of nebulized R6G aerosols on Au-TiO_2_ nanocomposites (Figure S3); SERS spectrum of SARS-CoV-2 spike proteins for various concentrations (Figure S4); SERS spectra of artificial respiratory aerosols (Figure S5); and SERS spectra of nebulized SARS-CoV-2 lysates in artificial respiratory aerosols for concentrations ranging from 0 to 10^4^ pfu/mL (Figure S6). The data that support the findings of this study are available upon reasonable request (PDF)
