# Supplementary material for: Highly Adsorptive Au-TiO2 Nanocomposites for the SERS Face Mask Allow the Machine-Learning-Based Quantitative Assay of SARS-CoV-2 in Artificial Breath Aerosols
Source: ACS Appl Mater Interfaces. 2022 Nov 30;14(49):54550–7. doi: 10.1021/acsami.2c16446 (PMC9718102; doi:10.1021/acsami.2c16446)
Supplement: Supplementary file 1 — am2c16446_si_001.pdf [file am2c16446_si_001.pdf]

Supporting Information

Highly Adsorptive Au-TiO_2 Nanocomposites for SERS Face Mask allow Machine-Learning based Quantitative Assay of SARS-CoV-2 in Artificial Breath Aerosols

Charles S. H. Hwang[1,3†], Sangyeon Lee[1†], Sejin Lee[1,3], Hanjin Kim[1], Taejoon Kang[2], Doheon Lee[1*], and Ki-Hun Jeong[1,3*]

[1] Department of Bio and Brain Engineering, Korea Advanced Institute of Science and Technology (KAIST), 291 Daehak-ro, Yuseong-gu, Daejeon, 34141, Korea.

[2] Bionanotechnology Research Center, Korea Research Institute of Bioscience and Biotechnology (KRIBB), 125 Gwahak-ro, Yuseong-gu, Daejeon 34141, Korea.

[3] School of Pharmacy, Sungkyunkwan University, Suwon, 16419, Korea

[4] KAIST Institute for Health Science and Technology (KIHST), Korea Advanced Institute of Science and Technology (KAIST), 291 Daehak-ro, Yuseong-gu, Daejeon, 34141, Korea.

*Corresponding author. Email: dhlee@kaist.ac.kr , kjeong@kaist.ac.kr

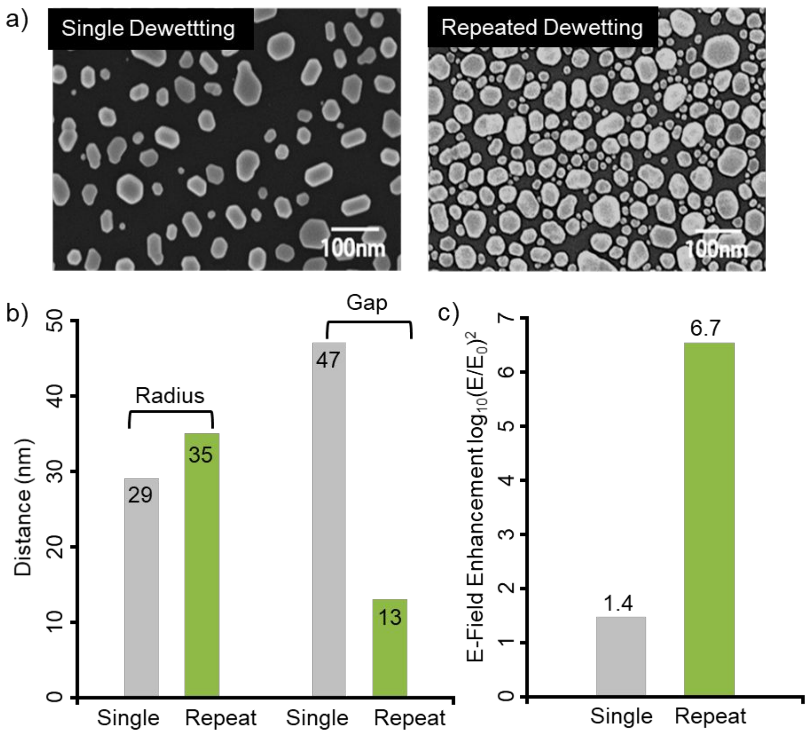

Figure. S1. (a) SEM images of Au nanoislands nanofabricated using single and repeated thermal dewetting of Au thin films. (b) Average radius and inter-particle distance of Au nanoislands fabricated using single and repeated dewetting. The average size is increased by 21%, and the interparticle distance is significantly reduced from 47 nm to 13 nm, exhibiting a greater number of plasmonic hotspots. The interparticle distance and the average radius was estimated assuming the nanoislands were arranged in a periodic square-lattice array with uniform size using ImageJ software. (c) The E-field enhancement of the Au nanoislands was calculated with the FDTD method. The average E-field enhancement is increased by approximately 5 times upon repeated thermal dewetting.

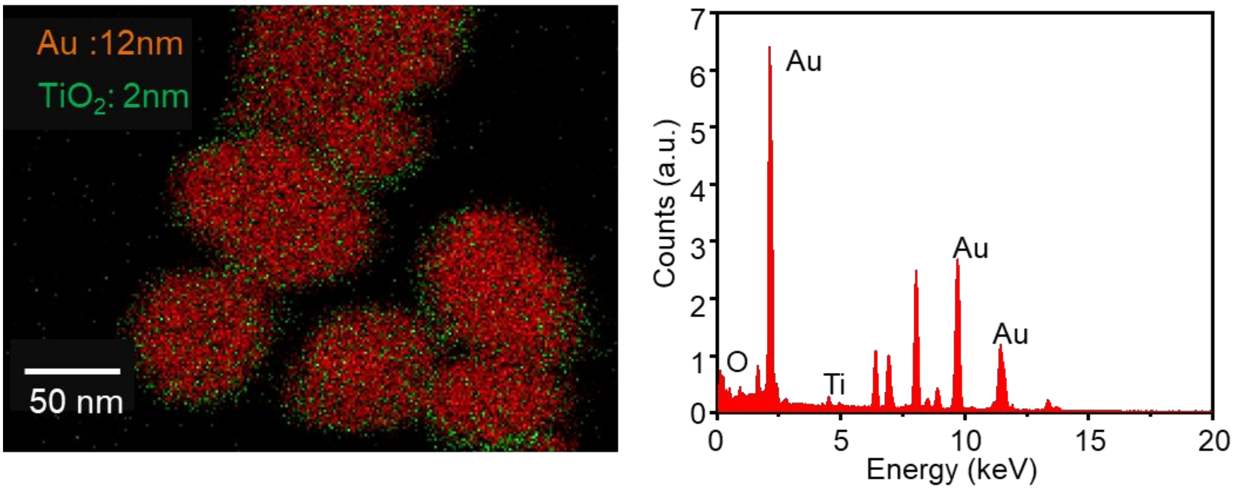

Figure. S2. The EDX elemental analysis confirms that the Au-TiO_2 nanocomposites with the atomic ratio of 83 % (Au) and 17 % (TiO_2) were successfully fabricated with respect to the initial target thickness of 12 nm (Au) and 2 nm (TiO_2). The 2 nm TiO_2 thin layer evaporated in Volmer-Weber mode is explicitly observable in the EDX image.

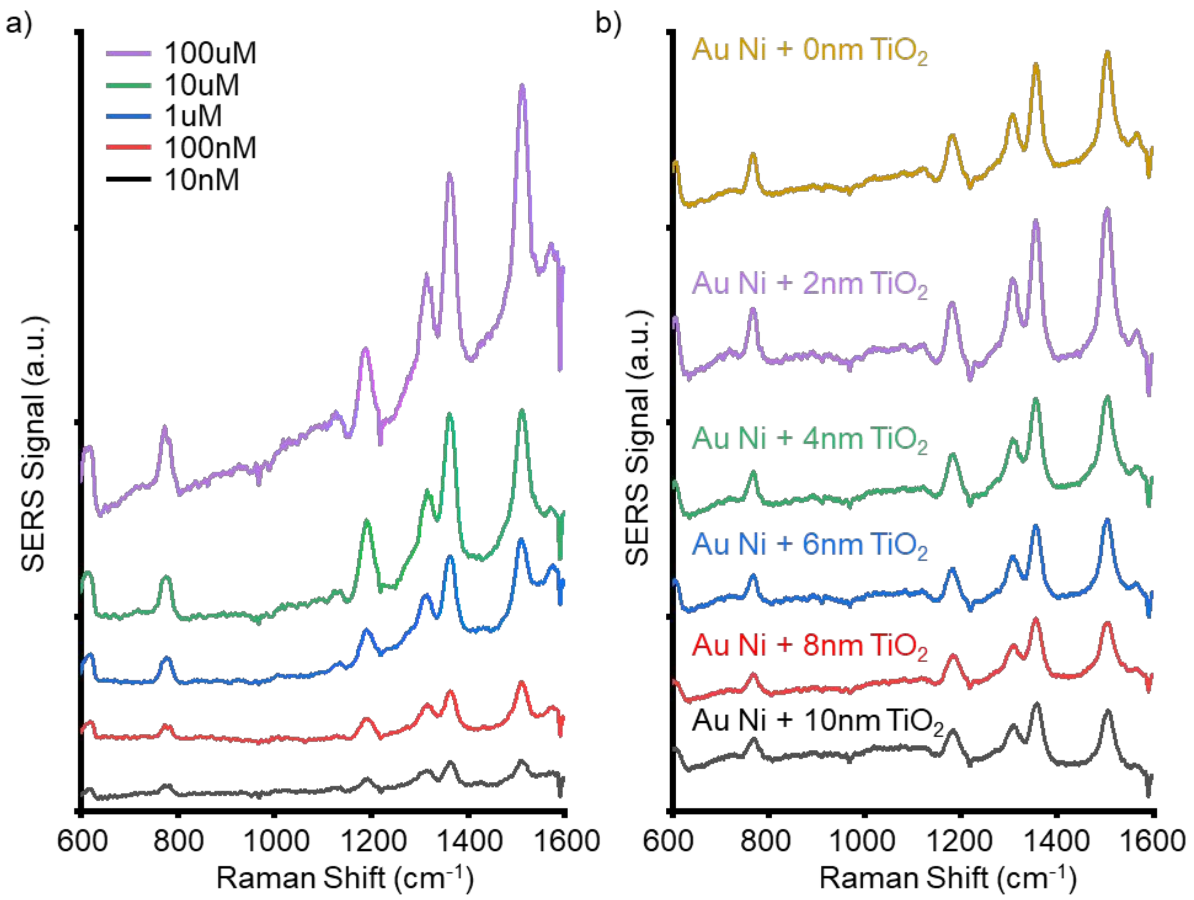

Figure. S3. SERS intensity of nebulized R6G aerosols on Au-TiO_2 nanocomposites depending on various (a) R6G concertation, and (b) TiO_2 thicknesses. Strong characteristic SERS peaks of R6G were successfully observed, including C-H in-plane at 773cm^{-1}, C-H out-plane at 1183cm^{-1}, C-C stretching at 1360$cm^{-1,}$ and C-C stretching at 1507cm^{-1}. The aerosol nebulization time was 10 seconds, and the SERS signals acquisition time was 1 second under the excitation of 633 HeNe laser beam.

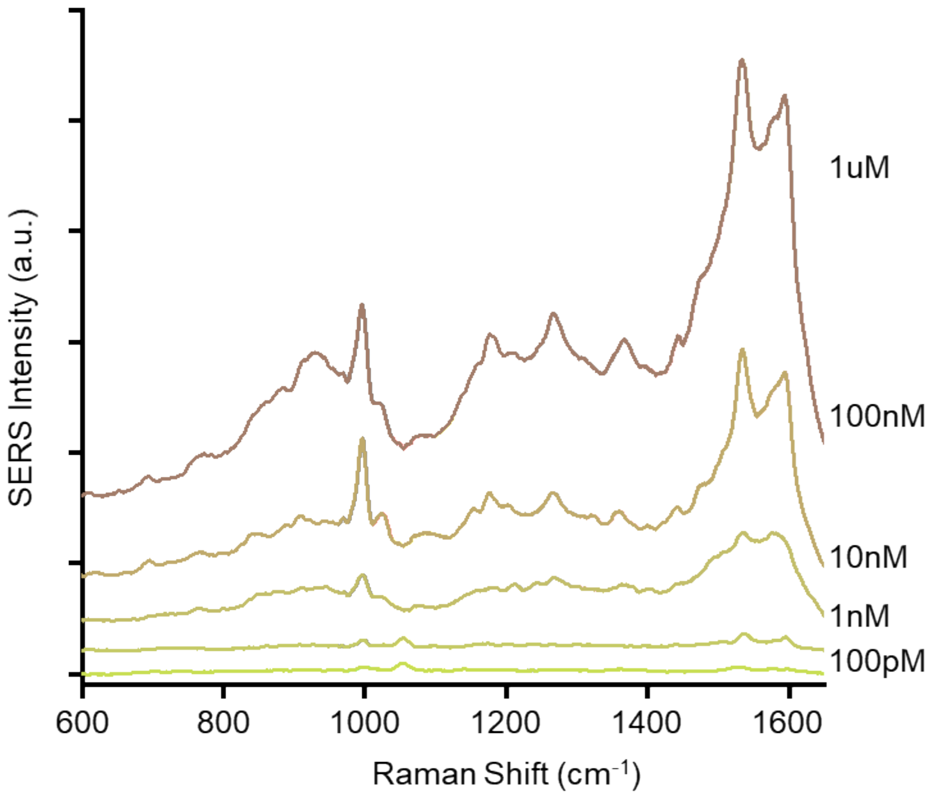

Figure. S4. SERS spectrum of SARS-CoV-2 spike protein for various concentrations. The Au-TiO2 SERS substrate exhibit extremely low-level concentration detection of SARS-CoV-2 spike protein at a 100pM level

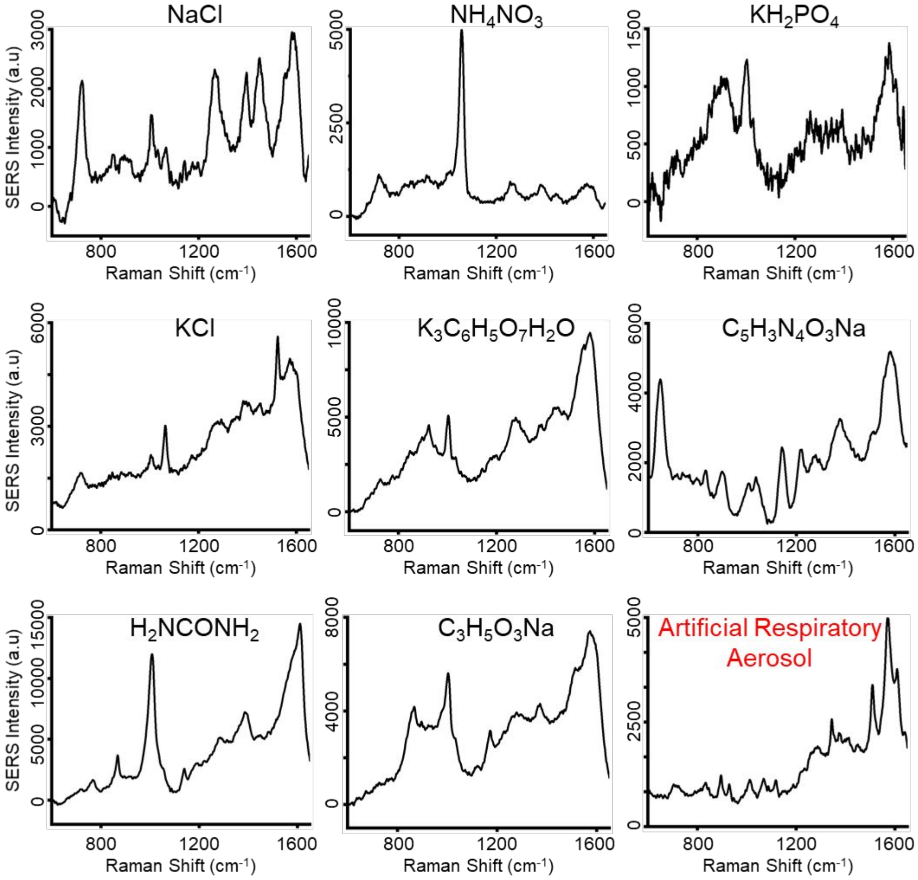

Figure. S5. The measured SERS spectra of artificial respiratory aerosol using Au-TiO_2 SERS substrate for individual chemical constituents. The acquisition time for the individual sample was 10 seconds, while that of the mixture was one second. The bottom right graph exhibits the SERS spectrum of the mixed artificial respiratory aerosol. Strong Raman bands appear around 1400-1600 cm^{-1} that significantly interfere with the Raman peaks of SARS-CoV-2 spike proteins.

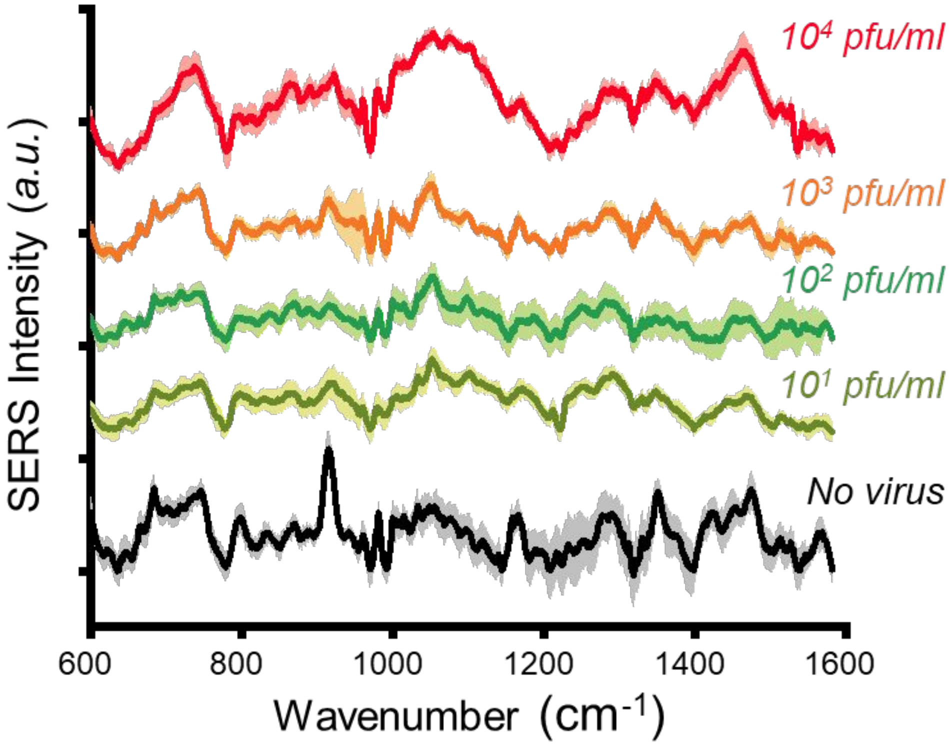

Figure. S6. The measured SERS spectra of nebulized SARS-CoV-2 lysates in artificial respiratory solutions for concentrations ranging from 0 to 10^4 *pfu/ml*. The SERS spectra were measured at five different points to evaluate the reproducibility and homogeneity of the Au-TiO_2 SERS substrates. The graph displays averaged SERS spectra (solid line) and their standard deviations (shadowed area). The average error, defined as the ratio of standard deviation to the average, was calculated as 10.5% for the Raman shift range of 600-1600 cm^{-1}.
